# Expression of *IMPDH* mRNA after Mycophenolate Administration in Male Volunteers

**DOI:** 10.1155/2014/870209

**Published:** 2014-07-01

**Authors:** Sollip Kim, Woochang Lee, Sail Chun, Tae Hyun Um, Won-Ki Min

**Affiliations:** ^1^Department of Laboratory Medicine, Ilsan Paik Hospital, Inje University College of Medicine, Goyang 411-706, Republic of Korea; ^2^Department of Laboratory Medicine, University of Ulsan College of Medicine and Asan Medical Center, 88 Olympic-ro, 43-gil, Songpa-gu, Seoul 138-736, Republic of Korea

## Abstract

*Background*. Mycophenolic acid (MPA) is the first-line antimetabolic immunosuppressants used in solid organ transplantation. Here, *in vivo* expressions of the pharmacodynamic marker *IMPDH* mRNA were analyzed to investigate its usefulness in assessing drug effects. *Materials and Methods*. Six healthy male volunteers who had the same genotype for genes known to be associated with drug metabolism and effects were selected to remove the confounding effect of these genotypes. Mycophenolate mofetil (MMF, 1 g) was administered once to each subject, and blood samples were collected with certain interval before and after MMF administration to measure lymphocyte expression levels of *IMPDH1* and *IMPDH2* mRNA. One week later, the experiment was repeated. *Results*. Whereas *IMPDH1* mRNA expression was stable, *IMPDH2* mRNA expression showed 2 peaks in the first week. Both *IMPDH1* and *IMPDH2* mRNA expression in the second week remarkably decreased from the first week. *Conclusion*. The temporary increase in *IMPDH2* mRNA expression in the first week might be due to a reactive reaction against the plasma MPA concentration. In the second week, the intracellular guanosine monophosphate might be depleted, rendering *IMPDH2* mRNA synthesis inactive. When MPA is regularly administered to reach a steady state, the *IMPDH2* mRNA expression may be kept low and may effectively reflect biological responses regardless of drug intake.

## 1. Introduction

Mycophenolate mofetil (MMF) is the first-line antimetabolic immunosuppressive agent [1] used in human solid organ transplantation. It is usually administered with calcineurin inhibitors (CNI) such as tacrolimus and cyclosporine [2]. After administration, MMF is quickly hydrolyzed to mycophenolic acid (MPA), which is its active metabolite. When MPA is combined with inositol monophosphate dehydrogenase (IMPDH) in a cell, the catalytic action of IMPDH is inhibited and inosine monophosphate (IMP) cannot be converted to xanthosine monophosphate (XMP). Therefore, intracellular guanosine monophosphate (GMP) becomes depleted over time. Consequently, DNA synthesis, which requires guanosine triphosphate (GTP), does not occur and cell proliferation is inhibited [3]. In cells, GTP is synthesized not only through the de novo pathway by IMPDH but also through an alternative pathway. However, since T- and B-lymphocytes can use only the de novo pathway, MPA selectively inhibits lymphocyte proliferation [3, 4].

Since MPA has a narrow therapeutic range and many side effects, therapeutic drug monitoring is useful for several conditions such as dual immunosuppressive therapy, reduced-dosage CNI therapy, CNI switch or withdrawal, recipients with high immunologic risk, delayed graft function, and altered gastrointestinal/hepatic/renal function [5]. However, the trough level does not reflect its pharmacodynamic effects, so the practical dosing guidelines based on drug concentration remain to be established [1, 5]. The target area under the curve (AUC) of the MPA blood concentration between 30 and 60 mg h/L remains acceptable therapeutic window [5–7]. However, this method requires repeated blood collection, so its clinical application is limited [5]. Although the limited sampling strategy showed good association to full AUC, it also requires at least 3 h stay for 3-time sampling and preexisting population pharmacokinetic model [5].

Therefore, a pharmacodynamic marker that can accurately reflect the biological effects of drugs on target cells must be developed. The activity of IMPDH, which is the intracellular target of MPA, showed limited association with clinical outcomes [5, 8–13]. High-performance liquid chromatography is the most widely used method of measuring IMPDH activity, but it is complicated and requires much time and effort. Moreover, its results vary so much that its clinical application is not widely accepted. Generally, the real-time reverse transcription- (RT-) PCR method is sensitive and can be used in clinical laboratories quickly and conveniently. So, we applied the real-time RT PCR method for* IMPDH1* and* IMPDH2* mRNA expression measurement.

The association of* IMPDH *mRNA expression with the clinical outcomes of MMF-treated kidney transplant patients has been reported [14], but no study has reported the* in vivo IMPDH* mRNA expression immediately after MMF administration to volunteers with same condition. We selected healthy volunteers who have same genotypes, gender, and race to reduce the effect of between-subject variability. Reasons for between-subject variability include differences in albumin, bilirubin and hemoglobin concentrations, renal and hepatic function, coadministration of cyclosporine, comorbidities, body weight, concomitant medication, time after transplantation, gender, race, and genetic polymorphisms in drug-metabolizing enzymes [5]. In this study, the* in vivo IMPDH1* and* IMPDH2* mRNA expressions were measured over 10 h after MMF administration to healthy people with same genotype using the real-time RT-PCR method to investigate the potential use of these two genes as pharmacodynamic markers.

## 2. Materials and Methods

### 2.1. Study Design and Healthy Volunteers

This study was approved by the local Institutional ethics committee. All subjects provided written informed consent. The study design is shown in Supplementary Figure 1 (see Supplementary Material available online at http://dx.doi.org/10.1155/2014/870209).

A total of 75 healthy male volunteers were selected for this study. The selection criteria were as follows: (1) 18- to 50-year-old healthy Korean male volunteers with (2) a body mass index of 18.5–29.9 kg/m^2^ and (3) past histories and physical examination and laboratory test results that revealed no abnormal findings. Candidates were excluded from this study when they were (1) hypersensitive to MMF or related drugs, (2) female, (3) administered with other medications up to 14 days before the study or during the study, (4) under the influence of alcohol since 2 days before the study or during the study, (5) suffering from any disease, including mental illness, (6) infected with AIDS or syphilis, positive for anti-HCV, or hepatitis B carriers, and (7) on an unusual diet (such as a low-sodium diet) for whatever reason. For the laboratory tests, the subjects' complete blood counts (hemoglobin, hematocrit, and red blood cell (RBC), white blood cell (WBC), differential, and platelet counts) and general blood chemistry (glucose, total protein, albumin, creatinine, AST, ALT, ALP, total cholesterol, total bilirubin, and direct bilirubin) were obtained.

The single nucleotide polymorphisms (SNPs) [15–25] of the genes (*UGT1A8*,* UGT1A9*,* UGT2B7*,* IMPDH1*, and* IMPDH2*) known to affect MPA metabolism and functions were investigated in the subjects. The volunteers who showed the same genotype were selected for removing the confounding effect by genotype. Six subjects were finally selected for the* IMPDH* mRNA expression study. The subjects swallowed (not chewed) 1 g of MMF tablet with water after fasting for 8 h, after which they resumed their regular diet. From 3 days before the study, the subjects' alcohol intake, smoking, and intake of other medications, greasy foods, and excessive water were restricted. Blood samples were collected at regular intervals over 10 h before and after the MMF administration (at the baseline and after 1, 2, 3, 4, 6, 8, and 10 h). The plasma total MPA concentrations and the* IMPDH1* and* IMPDH2* mRNA expression levels in the lymphocytes were measured. One week later, when the administered drug was supposed to have been removed from the blood, the whole study protocol including MMF administration and blood sampling was repeated (Supplementary Figure 1). The time interval between week 1 and week 2 for all six volunteers was the same.

### 2.2. Genotyping for SNPs Associated with MPA Pharmacodynamics and Pharmacokinetics

Based on a literature review on genotyping for SNPs associated with the pharmacodynamics and pharmacokinetics of MPA, 10 SNPs of the genes known to affect MPA metabolism and effects were selected [15–25]. The primers and probes that were used for the genotyping were designed in house using Primer3Plus, a web-based software (http://www.bioinformatics.nl/cgi-bin/primer3plus/primer3plus.cgi/). Using the gene-specific primer and the allele-specific TaqMan probe, real-time PCR was conducted. In each well of the 96-well microplates, 0.5 *μ*L of 2X TaqMan Universal PCR Master Mix (10 *μ*L), 40X SNP genotyping assay, and 2 *μ*L of template DNA were added and mixed after making the total volume up to 20 *μ*L with distilled H_2_O. The primers, probes, and master mix were purchased from Applied Biosystems (Poster, CA). The microplate was installed on a LightCycler 480 real-time PCR system (Roche Diagnostics, Indianapolis, IN) and allowed to react for 1 min at 95°C before 40 cycles of amplification were performed. Each cycle was comprised of 15 s denaturation at 95°C and 1 min extension at 50°C. The outcomes were read using the end-point genotyping method. Information on the primers and probes is shown in Supplementary Table 1.The dye for Reporter 1 was VIC, and the quencher was NFQ; for Reporter 2, the dye was FAM, and the quencher was NFQ.

### 2.3. Total MPA Blood Concentrations

Using ethylenediaminetetraacetic acid (EDTA) anticoagulated plasma, the EMIT 2000 Mycophenolic Acid Assay reagent, and the VIVA-E Drug Testing System (Siemens Healthcare, Deerfield, IL), the total and free MPA blood concentrations were measured according to the manufacturer's instruction. The analytical measurement range was 0.1–15 *μ*g/mL. The repeatabilities at the low, intermediate, and high concentrations were 3.4%, 3.6%, and 4.9%, respectively, and the total precisions were 6.1%, 4.5%, and 6.5%, respectively.

### 2.4. *IMPDH1* and* IMPDH2 *mRNA Expression Study

*(1) Lymphocytes Separation*. After the blood samples were collected, the monocytes were separated from the EDTA anticoagulated whole blood within 2 h. After 3 mL of Ficoll-Paque PLUS (Amersham Biosciences, Piscataway, NJ) was applied to the centrifuge tube, 6 mL of a solution that was a 1 : 1 mixture of phosphate buffer and whole blood was gently added to it to form a layer. Centrifugation was conducted at room temperature (18–20°C) at 400 g for 40 min. The upper layer was removed using a clean Pasteur pipette and the lymphocyte layer was carefully collected. To prevent contamination of the granulocytes and platelets, the sample was washed with 6 mL of phosphate buffer before it was centrifuged at room temperature at 100 g for 10 min. After the pellet was cleaned twice, it was put in a 1.5 mL guanidinium thiocyanate solution (RNA/DNA Stabilization Reagent for Blood/Bone Marrow; Roche Diagnostics, Mannheim, Germany) to be vortexed for 5 min, and then the test tube was slowly stirred for over 4 h.

*(2) RNA Extraction and cDNA Translation*. RNA was manually extracted from the lymphocyte suspension. The High Pure RNA Extraction Kit (Roche Diagnostics) was used according to the manufacturer's instructions. The concentration and purity of the extracted RNA were measured using a Nanodrop 2000 (Thermo Scientific, Wilmington, DE).

Within 1 h of the extraction, the RNA was translated into cDNA using AccuPower CycleScript RT PreMix dN6 (Bioneer, Daejeon, Republic of Korea) according to the manufacturer's instructions. Amplification was then done using a PTC-200 thermal cycler (MJ Research, Waltham, MA).

*(3) Development and Validation of Real-Time PCR for the IMPDH1 and IMPDH2 mRNA Expressions*. As an internal positive control, the* ACTB *gene that encodes *β*-actin was selected. Five primer sets of* IMPDH1, IMPDH2*, and* ACTB *were designed based on NM_000883.3, NM_000884.2, and NM_001101.3, respectively, using Primer3Plus. To prevent contamination by genomic DNA, at least one of the forward and reverse primers was placed at the exon-exon junction. For efficient real-time PCR, the size of the final product was limited to 150 nucleotides or less. Using the prepared primer, real-time PCR was conducted to select the primer set that had the least cycle threshold (Ct) and which showed a single peak in the melting curve analysis. Information on the selected primer is shown in Supplementary Table 2. With the selected primer, real-time PCR was conducted using the AccuPower Greenstar qPCR PreMix & Exicycler 96 Real-Time Quantitative Thermal Block (Bioneer) device. After 10 min cultivation at 95°C, a denaturation cycle at 94°C for 20 s with an extension at 54°C for 30 s was repeated 45 times. The standard DNAs of the three genes were synthesized at the concentrations of 2 × 10^0^, 2 × 10^1^, 2 × 10^2^, 2 × 10^3^, 2 × 10^4^, 2 × 10^5^, 2 × 10^6^, and 1 × 10^8^ to evaluate the linearity of the test. The precision was calculated through four repeated measurements.

*(4) IMPDH1 and IMPDH2 mRNA Expression Levels in the Volunteers*. Using the extracted RNA from the lymphocytes of the six subjects, the mRNA expression levels of the* IMPDH1, IMPDH2,* and* ACTB* genes were measured. Each batch included the standard DNA, and the samples were quadruplicated. The expression level was calculated using the standard curve method for relative quantification. It was described as arbitrary unit, showing the ratio of* IMPDH* mRNA expression to* ACTB *(internal control gene) mRNA expression. For the calculations and statistical analysis, Microsoft Office Excel 2010 (Microsoft, Redmond, WA) and MedCalc statistical software version 9.2.0.2 (MedCalc Software bvba, Ostend, Belgium) were used.

## 3. Results

### 3.1. Genotyping for SNPs Associated with MPA Pharmacodynamics and Pharmacokinetics

Nine (12%) of 75 volunteers had the same genotypes. Because three subjects refused to continue to participate in this study, 6 volunteers joined the* IMPDH* mRNA expression study. The genotypes of 6 volunteers were* UGT1A8 *rs1042597 CG,* UGT1A9 *rs17868320 CC, rs6714486 TT, rs72551330 TT,* UGT2B7* rs7439366 CC,* IMPDH1* rs2278293 AG, rs2278294 AG,* IMPDH2* rs121434586 CT, rs72639214 CC, and rs11706052 TT.

### 3.2. Total MPA Blood Concentrations

**Figure 1:**
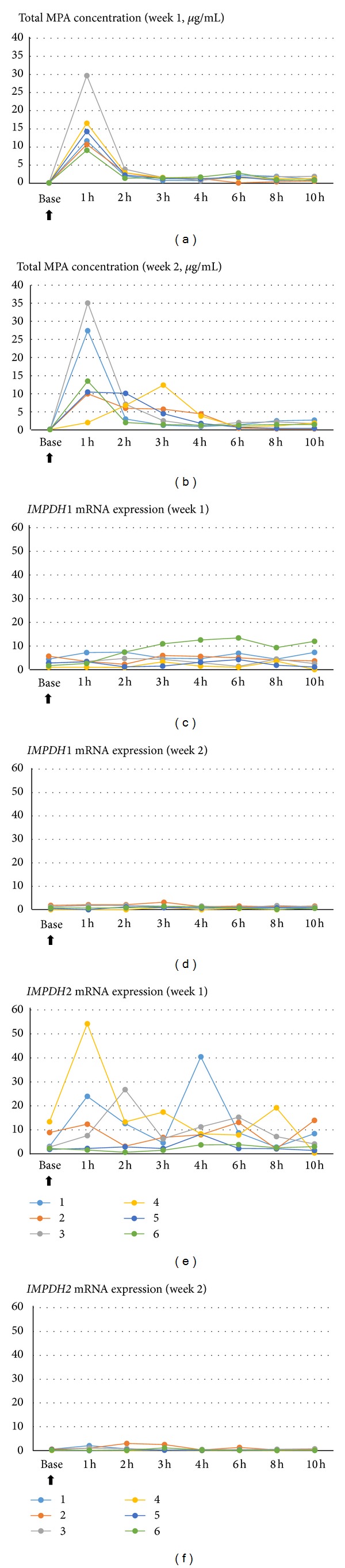
Plasma total MPA concentrations ((a) and (b)),* IMPDH1* mRNA expressions ((c) and (d)), and* IMPDH2* mRNA expressions ((e) and (f)) in the first week and the second week, respectively, over 10 h after the administration of MMF to the six volunteers. Black arrow means the administration of MMF. The *Y*-axis in mRNA expression graph is arbitrary unit, showing the ratio of* IMPDH* mRNA expression to* ACTB* (reference gene) mRNA expression.

The plasma total MPA level peaked 1 h after MMF intake and abruptly decreased 2 h later. The plasma concentrations of MPA of some subjects showed a second peak at 3–10 h after MMF intake, which was attributed to the enterohepatic circulation. The same pattern was seen when the experiments were repeated one week later (Figures 1(a) and 1(b)).

### 3.3. Validation of Real-Time PCR for the* IMPDH1* and* IMPDH2* mRNA Expressions

Linearity evaluation confirmed the linearity in all concentration ranges (2 × 10^0^–1 × 10^8^). All* R* ^2^ values of the regression lines were 0.99 or higher, showing excellent linearity. From the precision measurement, the mean Ct standard deviations of* ACTB, IMPDH1*, and* IMPDH2* were determined to be 0.44 (range, 0.01–5.59), 0.35 (0.01–1.59), and 0.11 (0.01–0.99), respectively.

### 3.4. *IMPDH1* and* IMPDH2* mRNA Expressions in the Volunteers

In the first week, the* IMPDH1* mRNA expression level did not change significantly according to the time interval, whereas the* IMPDH2* mRNA expression level peaked 1–3 h after drug intake. The level increased again 3–10 h after intake to show a double peak (Figures 1(c) and 1(e)). Expression levels of* IMPDH1 *and* IMPDH2* mRNA in the second week were remarkably lower than in the first week (Figures 1(d) and 1(f)).

## 4. Discussion

In the present study, 1 g (the typical unit dosage) of MMF was administered to volunteers to observe* IMPDH1* and* IMPDH2 *mRNA expression pattern and blood MPA concentrations. The observations were done at predefined intervals over 10 h before and after drug intake. The experiment including drug administration and samplings was repeated a week later to evaluate how fast mRNA expression decreases upon MMF intake. To exclude the influence of other factors on the mRNA expression, the study was limited to 18- to 50-year-old Korean healthy male volunteers. Ten genotypes that are known to affect the metabolism and biological effects of MMF were tested, and the subjects who had the same genotypes were selected for inclusion in the* in vivo* study. An association of* IMPDH* mRNA expression with clinical outcomes of MMF-treated kidney transplant patients has been reported [3, 14, 26, 27], but no study has been reported on the behavior of the* in vivo IMPDH* mRNA expression immediately after drug administration with the same condition.

The* IMPDH1* mRNA expression levels in the first week were stable, while the* IMPDH2* mRNA expression levels showed two peaks, at 1–3 h and 3–10 h after intake. The first* IMPDH2* mRNA expression peak is thought to be a temporary reactive reaction to the plasma MPA concentrations, and the second peak is thought to have been a response to the increase in blood MPA concentration caused by the enterohepatic circulation. In contrast, in the second week, the* IMPDH1 *and* IMPDH2* mRNA expression levels quite decreased compared with those of the first week (Figures 1(c)–1(f)). The possible reason is that* IMPDH1 *and* IMPDH2* mRNA synthesis was deemed to be inactive due to the depleted intracellular GMP. Given that intracellular MPA firmly combines with IMPDH enzyme and XMP through a hydrogen bond and the van der Waals force [28], MPA is thought to consistently inhibit GMP proliferation, thereby depleting GMP. In an* in vitro* experiment, when MPA was added after culturing of mixed lymphocytes, lymphocyte proliferation was inhibited for at least 72 h [3, 26, 27]. These outcomes showed that the inhibitive effect of MPA may be maintained for a considerable length of time, as long as guanosine is not provided from outside sources. Based on this, when MPA is taken on a regular basis to reach a steady state, intracellular GMP might become depleted, and consequently, the* IMPDH2* mRNA expression level may remain low. Therefore, one-point sampling test results may effectively reflect the biological responses regardless of the time of the drug intake, enabling its use as a marker. In contrast, the drug was removed from the blood within 8 h after intake in the first- and second-week experiments. Even though a steady state was attained after drug intake that lasted for longer than a month, the blood concentration level showed a pattern of an increase followed by a decrease with the passing of time. Accordingly, the AUC must be calculated for use as a surrogate marker. Some of the 6 volunteers showed the low first peak and second peak in week 1. Unfortunately, we do not know the exact reason of this result. However, if we have more sensitive technique, maybe we could see the peak in week 1.

In this study, the* IMPDH1 *and* IMPDH2* mRNA expression levels were measured via the real-time PCR method and SYBR Green. SYBR Green is an asymmetric cyanine dye that combines with double-stranded DNA, as well as with single-stranded DNA and RNA, though to a lesser extent. Even though SYBR Green has a lower specificity than the TaqMan probe, which combines only with a single helix that has a relevant base sequence, it has a high sensitivity level that allows it to be widely used to measure expression levels. Here, a combination that showed single peak was selected through fusion curve analysis that was repeated in each test to confirm the single peak and, accordingly, to secure the specificity of the test. As the standard DNA was synthesized for application, the linearity was confirmed at 8 log and the precision was excellent, with the standard deviations of the* IMPDH1, IMPDH2*, and* ACTB* gene Ct values at 0.5 or lower. Because real-time PCR is a convenient and straight-forward method that can provide results within 2 h after DNA extraction, it can also be widely used in clinical laboratories.

Because RBCs comprise a large portion of the whole blood cells and express IMPDH, the IMPDH expression of RBCs is most probably obtained when the test is conducted with whole blood. In this study, the lymphocytes were separated manually using the Ficoll-Hypaque technique. However, in clinical application, a commercial RNA stabilizing lymphocyte isolation blood tube may be used to achieve more stable outcomes.

One limitation of the current study is that MMF was administered to healthy volunteers only once a week, and then the pattern of the* IMPDH* mRNA expression was observed. Because MMF is an immunosuppressive agent that can have severe side effects, it could not be administered multiple times in a row to healthy volunteers. Further studies on the expression pattern of* IMPDH* mRNA, examining patients after repeated administrations, may be needed in the future.

In conclusion, the* in vivo IMPDH1* and* IMPDH2* mRNA expression levels in healthy volunteers after MPA intake were observed weekly in this study to confirm the possible use of the* IMPDH2* mRNA expression test as an effective pharmacodynamic marker of biological responses. Moreover, the real-time RT-PCR method that was developed and verified in this study showed a wide linearity range with excellent precision. The* IMPDH1 *and* IMPDH2* mRNA expression tests can also be conveniently conducted in clinical laboratories.

## Supplementary Material

To reduce other causes of variability, we performed a genotyping assay for 10 SNPs that are influenced by the drug in a total of 75 healthy male volunteers. Six of the volunteers with the same genotypic pattern were given a single dose of 1000 mg MPA.
Blood was sampled pre-dose and at 1, 2, 3, 4, 6, 8, and 10 h post-dose.
The experiments were repeated 1 week later using the same 6 volunteers.

## Abbreviations

AUC: Area under the curve
GMP: Guanosine monophosphate
GTP: Guanosine triphosphate
IMP: Inosine monophosphate
IMPDH: Inosine monophosphate dehydrogenase
MMF: Mycophenolate mofetil
MPA: Mycophenolic acid
RBC: Red blood cell
SNP: Single nucleotide polymorphism
WBC: White blood cell
XMP: Xanthosine monophosphate.
