# Supplementary material for: Expression of IMPDH mRNA after Mycophenolate Administration in Male Volunteers
Source: Biomed Res Int. 2014 Jul 1;2014:870209. doi: 10.1155/2014/870209 (PMC4101204; doi:10.1155/2014/870209)
Supplement: Supplementary file 1 — To reduce other causes of variability, we performed a genotyping assay for 10 SNPs that are influenced by the drug in a total of 75 healthy male volunteers. Six of the volunteers with the same genotypic pattern were given a single dose of 1000 mg MPA. Blood was sampled pre-dose and at 1, 2, 3, 4, 6, 8, and 10 h post-dose. The experiments were repeated 1 week later using the same 6 volunteers. [file 870209.f1.pdf]

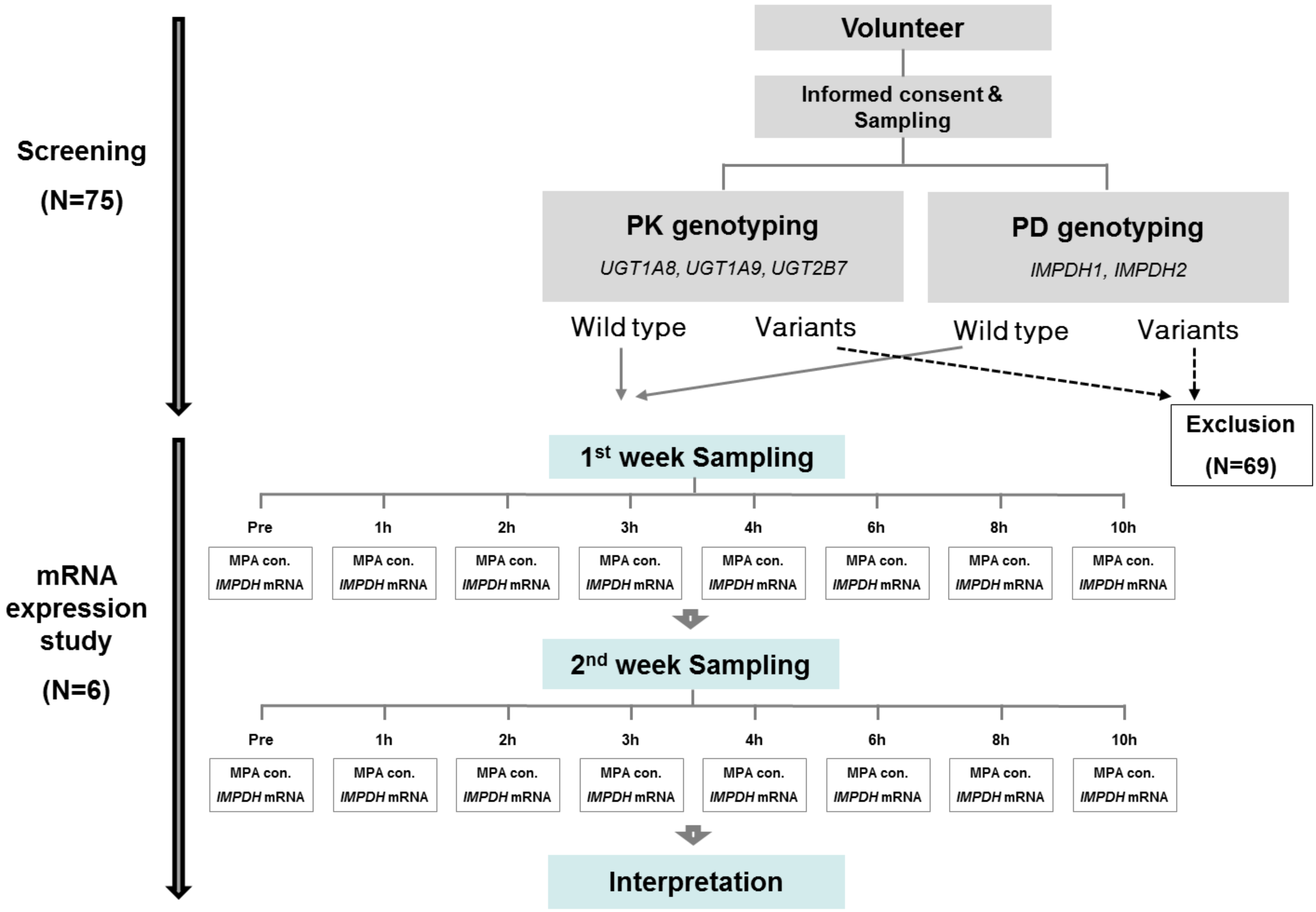

Supplementary Figure 1. Study design.

Supplementary Table 1. SNPs associated with MPA level and sequence information of primers and reporters for genotyping.

Gene	SNPs	Sequence Change	Forward Primer	Reverse Primer	Reporter 1	Reporter 2
UGT1A8	rs1042597	NM_019076.4: c.518C>G (*2)	CCCTCCCCTCTGTGGTCTT	GGCACTGTGCACCTTCTTCA	TAGTGGCAAGCTATTC	TAGTGGCAACCTATTC
UGT1A9	rs17868320	NM_021027.2: c.-2152C>T	TGATCTCAGCTCAATGCAACCT	TGCCTGTAATCCCAGCTACTCT	AGAATCACTTGAACCCGG	AGAATCACTTAAACCCGG
	rs72551330	NM_021027.2: c.98T>C	AGGCAGGGAAGCTACTGGTA	CCGACCTCATGGTGAACCA	CCCATCCGTGGGCAC	TCCCATCCATGGGCAC
	rs6714486	NM_021027.2: c.-275T>A	CTTCAAGGTCCAAAAGCATTGGTTA	GCCCTGTGCTGCAATGTT	ATAATTCTGCTACTAAACTT	ATTCTGCTTCTAAACTT
UGT2B7	rs7439366	NM_001074.2: c.802T>C (*2)	CTGACGTATGGCTTATTCGAAACTC	TGGAGTCCTCCAACAAAATCAACAT	AGTGGATGAGGAAACT	AGAGTGGATAAGGAAACT
IMPDH1	rs2278293	NM_000883.3:c.579+119G>A	GCCCTGCACTGCTACACT	AGGAGCCAGTGGGAAAATGAG	TCCTCTCAGTAGAGCCT	CCTCTCAGTGGAGCCT
	rs2278294	NM_000883.3:c580-106G>A	ATTTTCCCACTGGCTCCTCATC	CACAGGGAACAGAGGATGCA	AAGCCAGGATCCTCAG	AGCCAGGACCCTCAG
IMPDH2	rs121434586	NM_000884.2:c.787C>T	GCCCTGCACTGCTACACT	AGGAGCCAGTGGGAAAATGAG	TCCTCTCAGTAGAGCCT	CCTCTCAGTGGAGCCT
	rs11706052	NM_000884.2:c.819+10T>C	GCAGCTGCCCCTATTGC	GGTGTGGATGTAGTGGTTTTGGT	ACCTGTGTAGCAGCTC	CTGTGTGGCAGCTC
	rs72639214	NM_000884.2:c.98+93G>C (IVS1+93)	TTCCGAGAGCCGCCTTG	TGGCCAATTCCCGAAACCT	CCACGCGCACGCC	CCCACGCCCACGCC

Supplementary Table 2. Primer information for real-time PCR analysis of mRNA expression.

Genes	F/R	Sequences	Tm (°C)	Exon	Product Size (bp)
IMPDH1	F	TTCGTGCCCTACCTCATAGC	60.2	E15	80
	R	ATGGACCGAAGGACAGACAG	60.1	E15/16	
IMPDH2	F	CAGGCATCACACTGAAGGAG	59.4	E6	60
	R	GCAACTTTCCCTTCTTGCTG	60	E6/e7	
ACTB	F	CTCTTCCAGCCTTCCTTCCT	60	E4	116
	R	AGCACTGTGTTGGCGTACAG	60	E5	
